# miRNAs as Potential Biomarkers for Viral Hepatitis B and C

**DOI:** 10.3390/v12121440

**Published:** 2020-12-14

**Authors:** Dimitri Loureiro, Issam Tout, Stéphanie Narguet, Sabrina Menasria Benazzouz, Abdellah Mansouri, Tarik Asselah

**Affiliations:** Department of Hepatology, Université de Paris, CRI, INSERM UMR 1149, AP-HP Hôpital Beaujon, 92110 Clichy, France; dimitri.loureiro@inserm.fr (D.L.); issam.tout@inserm.tout (I.T.); stephanie.narguet@aphp.fr (S.N.); sabrina.menasria.benazzouz@gmail.com (S.M.B.); abdellah.mansouri@inserm.fr (A.M.)

**Keywords:** liver, inflammation, chronic liver diseases, chronic hepatitis, fibrosis markers, pro-fibrogenic, anti-fibrogenic, diagnosis

## Abstract

Around 257 million people are living with hepatitis B virus (HBV) chronic infection and 71 million with hepatitis C virus (HCV) chronic infection. Both HBV and HCV infections can lead to liver complications such as cirrhosis and hepatocellular carcinoma (HCC). To take care of these chronically infected patients, one strategy is to diagnose the early stage of fibrosis in order to treat them as soon as possible to decrease the risk of HCC development. microRNAs (or miRNAs) are small non-coding RNAs which regulate many cellular processes in metazoans. Their expressions were frequently modulated by up- or down-regulation during fibrosis progression. In the serum of patients with HBV chronic infection (CHB), miR-122 and miR-185 expressions are increased, while miR-29, -143, -21 and miR-223 expressions are decreased during fibrosis progression. In the serum of patients with HCV chronic infection (CHC), miR-143 and miR-223 expressions are increased, while miR-122 expression is decreased during fibrosis progression. This review aims to summarize current knowledge of principal miRNAs modulation involved in fibrosis progression during chronic hepatitis B/C infections. Furthermore, we also discuss the potential use of miRNAs as non-invasive biomarkers to diagnose fibrosis with the intention of prioritizing patients with advanced fibrosis for treatment and surveillance.

## 1. Introduction

Hepatitis B and Hepatitis C viral infections are still major public health problems of the 21st century despite the implementation of different therapeutics [1]. Around 257 million people are living with hepatitis B virus (HBV) and 71 million with hepatitis C virus (HCV) chronic infections [1]. Both HBV and HCV infections can induce liver complications such as cirrhosis and hepatocellular carcinoma (HCC). 

MicroRNAs (miRNAs) are small non-coding RNAs which regulate many processes in metazoans [2]. miRNAs expressions are frequently modulated by up- or down-regulation during fibrosis progression and cirrhosis. This review aims to summarize current knowledge of viral hepatitis B/C and miRNAs in the development and the progression of fibrosis. This review will also discuss the potential of miRNAs as biomarkers to diagnose fibrosis, since patients with advanced fibrosis are prioritized for treatment and surveillance.

## 2. Fibrosis Progression

Fibrosis is the consequence of chronic tissue injury and inflammation inflicted by various factors such as viral hepatitis, alcohol consumption, and non-alcoholic steatohepatitis [3]. Fibrosis process is characterized by an excessive and persistent accumulation of the extracellular matrix (ECM) as a consequence of activation of hepatic stellate cells, exaggerated expression of profibrogenic genes, and/or suppression of antifibrogenic genes [4]. The collagen deposits in the ECM and leads to the expansion of the portal zone with risk of cirrhosis and HCC development. Risk factors for fibrosis progression include host-related factors (advanced age, co-morbidities such as diabetes or obesity, etc.…) and exogenous factors (HIV co-infections, medication, and alcohol for example) [5]. Fibrosis regression in patients with HBV and HCV infection is achievable by antiviral treatments [6,7]. 

It is important to diagnose fibrosis and score its stage to prioritize patients for treatment. Two types of clinical tests are used to diagnose and determine the stage of fibrosis: non-invasive tests based on serological markers or on elasticity (fibroscan), despite their difficulties to differentiate mild from moderate fibrosis; and histological analysis after percutaneous liver biopsy. Among different scores used for liver fibrosis, METAVIR score is based on necro-inflammation and fibrosis evaluation [8]. Necroinflammation activity (A) is graded as A0 (absent), A1 (mild), A2 (moderate), or A3 (severe). Fibrosis is staged as F0, no fibrosis; F1, portal fibrosis without septa; F2, portal fibrosis with rare septa; F3, numerous septa without cirrhosis; F4, cirrhosis. Significant fibrosis is defined as METAVIR score F > 2 (F3 or F4) [8].

Complications are associated with percutaneous liver biopsy such as pain and bleeding [9]. Therefore, new non-invasive biomarkers are needed to determine, with high precision, the stage of fibrosis to improve prognosis evaluation in patients with Chronic Hepatitis B (CHB) and C (CHC). In patients with CHB, plasma HBsAg is used as a biomarker to stratify the risk of disease progression [10,11]. It has been shown that HBsAg titer was negatively correlated with the stage of the fibrosis in HBeAg-positive patients [11].

Circulating miRNAs are deregulated in liver fibrosis and HCC and are candidate biomarkers for diagnosis [12,13]. Diagnosis based on plasma miRNAs is presumably an attractive non-invasive strategy because of their stability and because of their potential correlation with different stages of fibrosis as reported in earlier studies [14].

## 3. HBV Infection

HBV is a small enveloped DNA virus belonging to the *Hepadnaviridae* family [15]. According to the World Health Organization (WHO), one third of people in the world have been exposed to HBV (antibodies to hepatitis B core antigen (anti-HBc)-positive) and around 257 million people are living with HBV chronic infection (Hepatitis B surface antigen [HBsAg]-positive) [1]. HBV is a hepatotropic virus, able to persist in infected cells and no current treatment is able to eradicate the virus from these cells [15]. 

**Figure 1:**
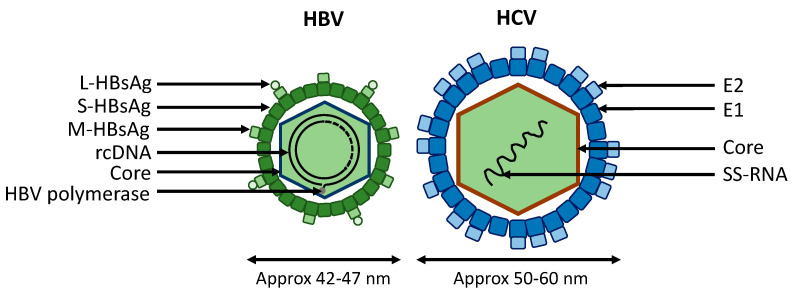
Comparison of hepatitis B virus (HBV) and hepatitis C virus (HCV) viral structures. The HCV virion is larger than the HBV virion by approximately 20 nm. HBV and HCV are two hepatotropic viruses which use different receptors for viral entry. Three different Hepatitis B surface antigens are exposed on HBV particles: the small (S-HBsAg), the medium (M-HBsAg) and the large (L-HBsAg) surface antigens. The HBV nucleocapsid is formed by dimers of hepatitis B core proteins (HBcAg) and contains a partly double-stranded DNA genome in relaxed conformation (3.2 kb in length). The HCV virion exposes two different viral envelope proteins on its surface: E1 and E2. HCV capsid is formed by HCV core proteins which contains a positive single-stranded RNA (9.6 kb in length). HBV = Hepatitis B virus; HCV = Hepatitis C virus; rcDNA = relaxed circular DNA; cccDNA = covalently closed circular DNA; SS-RNA = single stranded RNA.

An HBV infectious particle, which contains HBV genome (a partly double-stranded DNA in relaxed circular form called rcDNA), interacts with the human sodium taurocholate co-transporting polypeptide receptor (hNTCP or SLC10A1), the major HBV receptor described [16]. This interaction involved the Large Hepatitis B surface antigens (L-HBsAg), one of the three HBsAg exposed at the virion surface (Figure 1) [11]. Then, HBV rcDNA is release into the nucleus where it can be integrated into the human genome or repaired by different cellular mechanisms into covalently closed circular DNA (cccDNA) [17,18]. HBV cccDNA is a mini chromosome and is the major impediment to achieving an HBV cure and complete eradication of the infection [19]. Recent advances in treatment of CHB include nucleos(t)idic analogs with a high efficacy and favorable safety profile, but with long-life treatment duration [6]. However, while being able to control the viral replication, these antiviral therapies do not completely eliminate HBV in patients [6]. Furthermore, an effective prophylactic vaccine is available, but campaigns are not well-implemented. 

## 4. HCV Infection

HCV is an enveloped virus with a positive single-stranded RNA belonging to the *Flaviridae* family (Figure 1) [20]. In 2015, approximately 71 million people were living with HCV chronic infection worldwide, with 399,000 deaths due to liver complications (cirrhosis and HCC) [1]. The difference with HBV infection is that there is no HCV integration in the human genome and no viral reservoir because HCV replication is localized within the cytoplasm [7]. Drug discovery has allowed the development of HCV direct-acting antivirals with more than 95% of sustained virological response with complete eradication of HCV virus in infected patients and favorable tolerability [7]. The persisting problem is the access to diagnosis and to treatment, mainly in developing countries [21]. Moreover, no vaccine is available to prevent new infections and propagation of HCV [7]. 

## 5. Micro-RNA: A Small Non-Coding RNA

In the nineties, Victor Ambros and his team reported a microRNA (miRNA), a small non-coding RNA transcript from Lin-4 gene [22]. This miRNA Lin-4 controls the post-embryonic development of *c.elegans* by interacting with Lin-4 mRNA to regulate its translation [22]. miRNA are a class of endogenous single-stranded RNAs (approximately 20 nucleotides) which negatively regulate metazoans genes by targeting mRNAs in their 3′-untranslated region (3′-UTR) [2]. 

The interaction with the 3′-UTR of targeted mRNA induces the silencing of the gene by mRNA translational repression or degradation [2]. Because miRNAs regulate diverse cellular pathways or activities, their dysregulation is involved in liver fibrosis and a number of human cancers. The specific differences of miRNAs expression during CHB and CHC leads the way to their potential use in the diagnosis of fibrosis progression.

## 6. miRNAs in Normal Liver Tissue

The liver consists of various cell types (parenchymal hepatocytes, non-parenchymal biliary epithelial cells, lymphoid cells…). Each cell type expresses a unique miRNA profile. While miRNAs are up- or down-regulated in almost every stage of hepatic development, they accelerate or inhibit liver proliferation and play a major role in the regulation of diverse liver functions. It has been shown that a total of 277 miRNAs are expressed in the liver, with miR-122 being one of the most abundant and liver-specific miRNAs [23,24]. Besides miR-192, miR-199a/b-3p, miR-101, miR-99a, and let-7a/b/c/f (let-7 family), are abundant in liver whose miR-122 accounts for 70% of total liver miRNAs. Expression of miRNAs in the normal liver has been established by microarray systems and library sequencing [23]. The function of miR-122 has been explored in a variety of in vivo studies, including the miR-122 gene knockdown or silencing of miR-122 with antagonists. Mir-122 is an anti-inflammatory and anti-tumorigenic effector in liver [25]. In the miR-122 gene knockdown mice, it has been shown that it acts as a key regulator of cholesterol and fatty-acid metabolism; and its gene resulted in the development of liver tumors [24]. Results of studies evaluating up- and down-regulated miRNA profiles in differentiating liver cells are not consistent. Besides, various technical issues, including differences in clinical samples, different miRNA matrices in miRNA assays, and different degrees of miRNAs expression among studies suggest that the miRNA profile is also influenced by the origin of the progenitor cell and that it is difficult to compare miRNA profiles in different cellular developmental stages. 

## 7. Hepatitis B Genome Encodes for Two Viral miRNAs: HBV-miR-2 and 3

To promote their replication and gene transcription, and to control host genes expression, viruses have developed diverse strategies such as viral miRNAs production [26]. 

**Figure 2:**
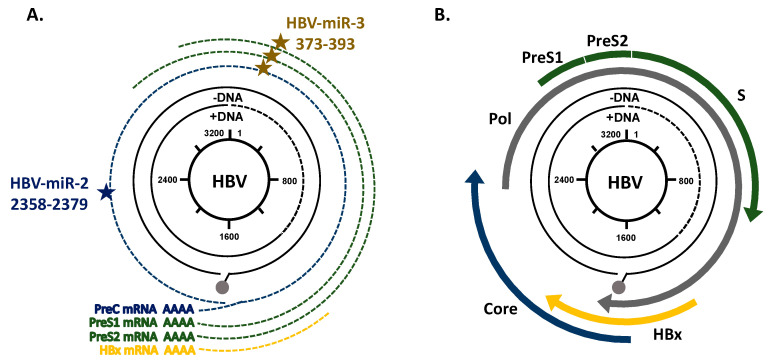
HBV genome organization. (**A**) Different HBV transcripts and positions of HBV sequences encoded for HBV-miR-2 and HBV-miR-3. HBV-miR-2 (blue star) extended from nucleotides 2358 to 2379 of HBV pgRNA. HBV-miR-3 (brown stars) is encoded in the HBV genome at the position 373 to 393 in PreC, PreS1, and PreS2 mRNAs. (**B**) The HBV genome contains four overlapping Open Reading Frames (ORFs): core proteins, HBV polymerase, Surface proteins, and Hepatitis B X protein (HBx).

The HBV 3.2 kp partly double-stranded DNA contains four overlapping open reading frames (ORFs) (Figure 2). Five viral transcripts are produced by the human RNA polymerase II which are translated into seven HBV proteins: PreC mRNA (3.5 kb), pre-genomic RNA (pgRNAs, 3.5 kb), PreS1 mRNA (2.4 kb), PreS2 mRNA (2.1 kb), and X mRNA (0.7 kb) (Figure 2A) [15]. Additionally, recent studies highlighted that in addition to viral transcripts, HBV produced two different miRNAs: HBV-miR-2 and HBV-miR-3 [27,28].

### 7.1. HBV-miR-2

**Figure 3:**
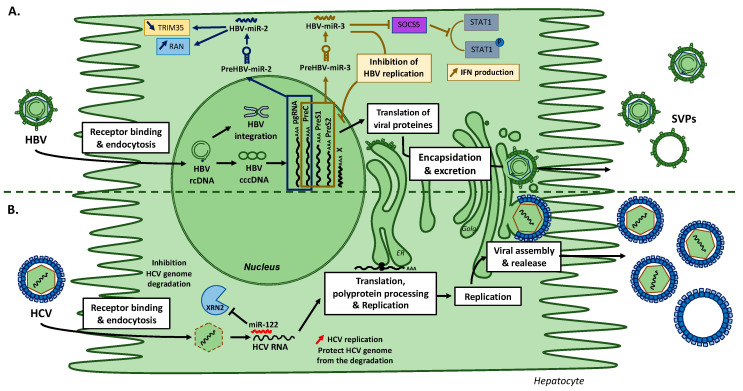
HBV-encoded miRNAs production and their targets (**A**), and modulation of HCV replication by miR-122 (**B**). (**A**) HBV encodes for two different miRNAs: HBV-miR-2 and HBV-miR-3. HBV-miR-2 is encoded from HBV pgRNA and HBV-miR-3 from PreC, PreS1 and PreS2 mRNAs. HBV-miR-2 down-regulates TRIM35 and up-regulates RAN expressions. HBV-miR-3 represses SOCS5 and PPM1A expression to increase interferons. Moreover, HBV-miR-3 regulates HBV core proteins (HBc) and the level of pgRNA. (**B**) Mode of actions of miR-122 on HCV replication and maintenance. miR-122 binds to HCV RNA genome and protect HCV against the degradation by exoribonucleases and promote HCV replication. HBV = Hepatitis B virus; HCV = Hepatitis C virus; TRIM35 = Tripartite motif-containing protein 35; PPM1A = Protein phosphatase 1A; RAN = Ras-related Nuclear protein; rcDNA = relaxed circular DNA; cccDNA = covalently closed circular DNA; SOCS5 = Suppressor of cytokine signaling 5; STAT1 = Signal transducer and activator of transcription 1; SVPs = Subviral Particles; XRN2 = 5′-3′ exoribonuclease 2.

Yao et al. identified HBV-miR-2 by deep sequencing [27]. HBV-miRNA produced from the pgRNA is encoded by the sequence extended from the nucleotides 2358 to 2379 of HBV genome (Figure 2A) [27]. HBV-miR-2 is expressed in infected livers and secreted in serums of patients with HBV infection and in those with HBV-related HCC. The HBV-miR-2 sequence is rather well preserved among different HBV subtypes, with only a single nucleotide change in the subtypes D, G and H. Few is known however about the role and implication of HBV-miR-2 in HBV replication and in liver diseases. HBV-miR-2 may act as an oncogene and promotes cell growth, migration, and invasion during HCC. HBV-miR-2 down-regulates tripartite motif-containing protein 35 (TRIM35) and up-regulates ras-related nuclear protein (RAN) expressions in vitro (Figure 3A) [27]. In ovarian cancer and HCC, modulation of RAN expression is correlated with cells proliferation, migration, and invasion [27,29].

### 7.2. HBV-miR-3

HBV-miR-3 is encoded from nucleotides 373 to 393 in the HBV genome and is generated from three HBV transcripts: PreC, PreS1, and PreS2 mRNAs (Figure 2A) [28]. During HBV infection, HBV-miR-3 is highly expressed and its expression correlates with HBV activity [30]. HBV-miR-3 enhances IFN production, activates JAK/STAT signaling, affects macrophages polymerization/depolarization, and induces the production of interferons and IL-6 by repressing SOCS5/STAT1 pathway (Figure 3A) [31]. Moreover, HBV-miR-3 interacts directly with the Protein phosphatase 1A (PPM1A) and Phosphatase and TENsin homolog (PTEN), silences these human genes, and enhances cell invasion and proliferation in HCC development [30,32]. Furthermore, HBV attenuates its replication targeting its HBV 3,5 kb mRNA transcript with HBV-miR-3 to reduce the expression of HBV core proteins (HBc) and the level of pgRNA [28]. 

The role of HBV-miR-2 or HBV-miR-3 in liver fibrosis remains unclear. These two miRNAs encoded by HBV could be considered as potential targets to block HBV replication and thus the comprehension of their mechanisms of action needs to be further investigated. 

## 8. HCV Replication Is Modulated by miR-122 Expression

**Figure 4:**
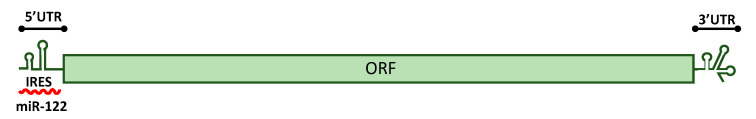
miR-122 and interaction with HCV RNA. HCV genome is a positive single-stranded RNA particle which encodes for a polyprotein processed into ten different proteins. miR-122 is essential for HCV replication. miR-122 binds the 5′-UTR, the IRES region, protects against the degradation of HCV genome, and thus promotes HCV replication. IRES = Internal Ribosome Entry Site; 3′-UTR = 3′-Untranslated Transcribed Region; 5′-UTR = 5′-Untranslated Transcribed Region; ORF = Open Reading Frame.

HCV genome is a 9.6 kb positive single-stranded RNA with a single ORF (Figure 4) [33]. HCV encodes for a polyprotein processed into ten proteins using cell machinery [34]. HCV does not encode for viral miRNA. Highly expressed in liver tissue, miR-122 is encoded on the human chromosome 18 and is one of the liver specific miRNAs implicated in fatty-acid metabolism and acts as a tumor suppressor [25,35]. Interestingly, HCV needs miR-122 for its replication [36]. miR-122 binds the 5′-UTR, the Internal Ribosome Entry Site (IRES) region of HCV RNA genome and this interaction is essential for HCV replication (Figure 4) [37,38,39]. miR-122 inhibits the interaction of exoribonuclease 2 (XRN2) with HCV 5′UTR, and thus protects the viral genome from its degradation by XRN2 (Figure 3B) [40,41,42]. Targeting this interaction has been shown as a potential antiviral strategy to induce HCV genome degradation by inhibiting miR-122 interaction with HCV genome [43]. A miR-122 antagonist combined with direct acting-antivirals (DAAs) has been tested in cell culture. The results from these in vitro studies suggested the potential therapeutics of the miR-122 antagonist in order to treat patients not responding to DAAs treatment [43]. However, a virologic rebound was observed after several weeks of treatment suggesting the failure of the treatment [44]. This strategy for HCV infection has been stopped because of the success of direct-acting antivirals.

## 9. miRNAs and the Immune System during Viral Hepatitis

Multiple miRNAs modulate the immune system at different levels such as the differentiation of innate and adaptive immune cells [45]. During CHB, several immune components are dysregulated, and HBV may interact with miRNAs to modulates the immune system [46]. Examples of miRNA-influenced inflammation include miR-145, -148a, -200b, -200c, and -455 [47]. Up-regulated miR-181a regulates inflammatory responses by down-regulating IL-1a levels [48]. HBV can inhibit intrinsic RIG-I and RIG-G immune signaling via inducing miR-146a [49]. Furthermore, Singh et al. identified a deregulated network of miRNAs-mRNAs in DCs that seems responsible for an impaired immune response during HBV pathogenesis [50]. Regarding adaptive immunity, several miRNAs such as miR-155, -17–92, -181a, and -21 have been linked to B and T cell activation and differentiation as well as Treg activity [51]. miR-155 expression positively correlates with T-cell activation in CHB patients and is a potential biomarker for immune activation and disease progression in HBV infection [52]. miRNA regulation of the immune response and signaling has also been described in HCV infection [53].

A deeper understanding of the correlation between miRNA regulation and the immune response in CHB may lead to using candidate miRNA to manipulate these interactions as a potential therapeutic option.

## 10. miRNAs Expression during CHB and CHC Related Fibrosis

miRNAs may play pro-fibrogenic or anti-fibrogenic roles depending on cellular contexts. The reported changes in the expression of miRNAs modulation during HBV and HCV infection are presented in Table 1.

### 10.1. Dysregulation of Anti-Fibrogenic miRNAs in CHB

#### 10.1.1. miR-29

Recent studies have shown that miR-29 negatively regulates mRNAs encoding for proteins of the ECM. The downregulation of the ECM proteins such as collagen type I alpha 1 and 2 (COL1A1 and A2), collagen type III alpha 1 (COL3A1), elastin (ELN) and fibrillin 1 (FBN1) by miR-29 is leading to its anti-fibrotic activity [82]. Four forms of miR-29 have been described in humans and differ by two or three bases. miR-29b-1 and miR-29a are encoded by a human gene located on chromosome 7q32.3 and miR-29b-2 and miR-29c on chromosome 1q32.2 [54]. miR-29 expression is significantly modulated in fibrotic disorders, particularly in HBV infection. In fact, in patients with HBV infection, serum levels of miR-29 were significantly decreased according to fibrosis progression, necro-inflammation grades and in HBV-related-HCC [55,56,57]. Serum miR-29 levels were positively correlated with HBV DNA in patients with CHB [83]. Hepatitis B virus X protein (HBx) increases miR-29a expression in the HepG2 model inducing cell migration through the PTEN gene [84].

#### 10.1.2. miR-122

In the serum of patients with CHB, miR-122 expression was up-regulated at least 1,5 fold and was down-regulated in patients with HBV-related HCC [60,61,62,63,85]. miR-122 levels were negatively correlated with HBV DNA, ALT and HBsAg levels in CHB infection [86,87]. 

#### 10.1.3. miR-143

miR-143 is a tumor suppressor encoded by chromosome 5q32 and expressed in several tissues but down-regulated in various tumors [66]. Bao et al. investigated miR-143 expression in patients with CHB and showed a down-regulation (at least 3) were compared with the healthy control group [55]. Then, miR-143 expression was down-regulated in patients with CHB and correlated with the progression of the fibrosis [55]. This down-regulation was observed, as well, in patients with HBV-related-HCC [67].

#### 10.1.4. miR-185

miR-185 is encoded in Tango2 gene sequence in the intron region of chromosome 22q11.2. miR-185 expression was increased in serum from patients with HBV infection and positively correlates with liver fibrosis progression but no difference has been shown with HBV viral load [70]. When patients with HBV-related HCC were compared with patients with HBV infection and without HCC, a significant over-expression was observed for miR-185 in the plasma [71]. In vitro, expression of miR-185 induces a decrease of HBV activity/replication through targeting ELK1 in HCC cells [88]. Finally, miR-185 targets protein kinase C eta (PRKCH), affects viral replication and inhibited HBsAg expression [89]. 

### 10.2. Dysregulation of Pro-Fibrogenic miRNAs in CHB

#### 10.2.1. miR-21

miR-21 is highly expressed at the onset of fibrosis and is involved in many cancers such as breast cancer and digestive/gastric tract cancers (stomach, prostate, colon, pancreas). The gene coding for mirR-21 is located within the intronic region of TMEM49 human gene at chromosome 17q23.1. miR-21 targets many cellular genes, inhibiting tumor suppressor pathways, affecting collagen synthesis and activating hepatic stellate cells (HSCs) leading to fibrogenesis pattern [73]. In a cohort of patients with CHB from China with or without HCC-related to HBV, Qi and al. identified a down-regulation of serum levels of miR-21 in patients with CHB when compared to healthy control group. When miR-21 expression in patients with CHB were compared to patients with HBV-related HCC, no significant change could be observed suggesting that there is no difference of miR-21 expression in patients with CHB *versus* those with HCC [74]. In patients with CHB, expression of miR-21 in the serum significantly decreases as fibrosis progressed from no, minimal, and moderate fibrosis F0–F2 to advanced fibrosis and cirrhosis F3–F4 [55]. It has been reported that HBx induces miR-21 expression which enhances cell transformation and proliferation during HCC [90,91].

#### 10.2.2. miR-221 and miR-222

miR-221 and miR-222 are encoded by human chromosome X but are encoded in tandem. Depending on the cellular context, miR-221/222 may act as oncogenes or onco-suppressor genes. In numerous types of human cancers, miR-221 and miR-222 have been shown to be over-expressed and in particular in HCC [77]. miR-221 and 222 levels expressions were compared in serums and in liver biopsies in patients with CHB and CHC. Expression of liver miR-222 was positively correlated with the progression of the fibrosis; however serum levels were unchanged during fibrosis progression in patients with CHB. miR-221 expression was not affected during fibrosis progression in liver biopsies and serums of patients with CHB [14]. Chen et al. demonstrated that miR-221 was over-expressed in HBx-transfected cells [92].

#### 10.2.3. miR-223

miR-223 is encoded by q12 locus of X chromosome in humans and is involved in the regulation of several transcription factors. Its pathogenicity has been described in liver diseases through interacting with the inflammatory signaling pathways and its pro-fibrotic effects by modulating inflammation [93,94]. Serum levels of miR-223 negatively correlated with fibrosis progression in patients with CHB [55,78]. In HBV-related HCC, miR-223 was significantly decreased in cancerous tissues in comparison to HBV non-cancerous tissues and the tumor size is negatively correlated with miR-223 serum levels [79]. In HepG2 transfected HBx cells, HBx down-regulates miR-223 and plays a crucial role in cell proliferation of HCC [95]. More studies are needed to better understand the associated mechanism. 

### 10.3. Dysregulations of Anti-Fibrogenic miRNAs in CHC

#### 10.3.1. miR-29

Serum levels of miR-29 are not affected in patients with CHC infection [58]. Interestingly, in people who inject drugs (heroin users) with HCV infection, levels of miR-29a, b, and c were significantly increased in comparison to healthy controls and patients with HCV infection [58]. However, in patients with HIV/HCV co-infection, a down-regulation of serum levels of miR-29a was shown when compared to healthy control group and patients with HIV infection [59]. In vitro, HCV decreases the expression of miR-29 activating HSCs and miR-29c has been shown to repress HCV replication targeting STAT3 and induce the production of IFN I [96,97].

#### 10.3.2. miR-122 

It has been reported that miR-122 promotes HCV replication and HCV depends on miR-122 for its viral cycle (Section 7). In patients with CHC, miR-122 levels were down-regulated and were negatively correlated with fibrosis stage and cirrhosis [14,64]. miR-122 expression was significantly lower in CHC-HCC group when compared to CHC group [65]. miR-122 levels in plasma of CHC were not correlated with fibrosis but positively correlated with inflammatory activity and finally increased in patients with HCV-related HCC [72,98].

#### 10.3.3. miR-143

miR-143 expression was found down-regulated in the serum of patients with HCV-related HCC than in healthy control group [68]. Furthermore, miR-143 serum levels were significantly lower in non-cirrhotic patients with CHC when compared to healthy controls [68]. Then, there is a positive correlation between miR-143 serum levels and fibrosis stages [69].

#### 10.3.4. miR-185

miR-185 was significantly decreased in patients with HCV-related HCC [72]. Furthermore, miR-185 acts as a negative regulator of host metabolism and lipid metabolism and appears to be crucial for HCV replication. miR-185 expression was down-regulated by HCV core protein during HCV infection to promote its replication [99,100].

In brief, miR-29, miR-143, miR-21 and miR-223 expressions decreased during CHB infection, while miR-122 and miR-185 expressions are increased during fibrosis progression in patients with CHB, miR-221 and miR-222 serum levels remain unchanged during CHB. In patients with HBV-related HCC, miR-185 expression was increased whereas miR-29, -122, -143 and miR-223 expressions were decreased. 

### 10.4. Dysregulations of Pro-Fibrogenic miRNAs in CHC

#### 10.4.1. miR-21

During CHC, levels of circulating miR-21 were unchanged during the course of the infection and increased in patients with HCV-related HCC [75,76]. In vitro, HCV upregulates miR-21 expression, promoting its replication, by suppressing type I IFN production [101].

#### 10.4.2. miR-221 and miR-222 

Expression of miR-222 in the liver and serum was lower in patients with CHC when compared to patients with CHB [14]. Furthermore, there is no difference in miR-221 expression in patients with CHC *versus* those with CHB. miR-221/222 hepatic and serum levels were unchanged during HCV infection [14].

#### 10.4.3. miR-223

The kinetics of miR-223 expression in patients with HCV infection are particularly different from HBV. In fact, miR-223 levels were increased in patients with advanced fibrosis (F3) and cirrhosis when compared to patients with no, minimal, and moderate fibrosis (F0–F2) [80]. miR-223 was significantly decreased in HCV-related HCC when they compared these patients with HCV and control group [81].

To summarize, in the serum of patients with CHC, during fibrosis progression, miR-143 and miR-223 expressions increased, while miR-122 expression decreased. miR-29, -21, -221 and miR-222 expressions were not affected during fibrosis progression and HCC development in patients with CHC. In HCV-related HCC, miR-122, -185, and miR-223 were significantly decreased.

## 11. Conclusions 

HBV and HCV infections remain major medical needs worldwide with around 330 million patients with CHB or CHC. Both HBV and HCV infections can lead to liver complications such as cirrhosis and HCC. Identifying patients, in whom fibrosis will progress rapidly, is crucial for disease prognosis and patient’s management, since patients with advanced fibrosis must be prioritized for treatment and screened for HCC.

**Figure 5:**
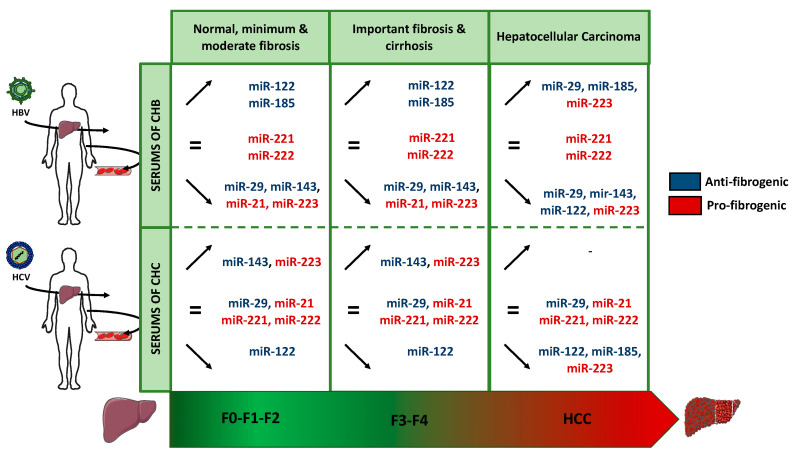
miRNAs dysregulated during fibrosis progression and HCC development in HBV and HCV infections. Expression increase (↑) decrease (↓) or its equal (=) during the two different mean stages of fibrosis progression mild and moderate fibrosis (F0 to F2), advanced fibrosis and cirrhosis (F3–F4) and HCC. The miRNA function as anti-fibrogenic and pro-fibrogenic is marked by blue and red color respectively.

Cellular miRNAs contribute to HBV and HCV pathogenesis by direct or indirect interactions with viral genome or proteins and molecules critical for the regulation of host or viral genes. Regulation of miRNAs expression upon HBV and HCV infections significantly differs between these viruses. In this review, we summarized in Figure 5 and Table 1, pertinent data showing the importance of miRNAs in the regulation of liver fibrosis and in inflammation processes in CHB and CHC. Many miRNAs are deregulated during CHC and CHB infections and are potential candidates for diagnosis and prognosis in such conditions. 

Due to an extensive number of miRNA targets and other clinical factors considered in a significant number of studies published so far, efforts should be made to establish specific and reproductive easy methods to identify reliable panels of miRNA biomarkers for early diagnosis and treatment of HBV- and HCV-related liver complications. The application of novel techniques such as next-generation sequencing, development of synthetic small RNAs, and hepatoma cell lines will impact the subsequent advances in miRNA studies related to HBV and HCV pathogenesis as well as miRNA deregulation in other pathological conditions. Clearly, further studies are warranted to confirm miRNAs potential in CHB and CHC patients. 

## Figures and Tables

**Table 1:** Anti and Pro-Fibrogenic miRNAs synthesis. Expression increase (↑) decrease (↓), it is equal (=) or not determined (Nd).

			Chronic Hepatitis B			Chronic Hepatitis C			Refs
	miRNAs	Chr	F0-F1-F2	F3-F4	HCC	F0-F1-F2	F3-F4	HCC	
Anti-Fibrogenic	miR-29	7q32.3; 1q32.2	↓	↓	↓	=	=	=	[54,55,56,57,58,59]
	miR-122	18	↑	↑	↓	↓	↓	↓	[14,60,61,62,63,64,65]
	miR-143	5q32	↓	↓	↓	↑	↑	nd	[55,66,67,68,69]
	miR-185	22q11.2	↑	↑	↑	nd	nd	↓	[70,71,72]
Pro-Fibrogenic	miR-21	17q23.1	↓	↓	=	=	=	=	[55,73,74,75,76]
	miR-221	X	=	=	=	=	=	=	[14,77]
	miR-222	X	=	=	=	=	=	=	
	miR-223	Xq12	↓	↓	↓	↑	↑	↓	[55,78,79,80,81]
